# Value of “Three Dimensional Multidetector CT Hysterosalpingography” in Infertile Patients with Non-Contributory Hysterosalpingography: A Prospective Study

**Published:** 2017

**Authors:** Shuchi Bhatt, Murtaza Sumbul, Rajpal Rajpal, Gita Radhakrishnan

**Affiliations:** 1- Department of Radio-Diagnosis, University College of Medical Sciences, Delhi University, New Delhi, India; 2- Guru Teg Bahadur Hospital, New Delhi, India; 3- Department of Obstetrics and Gynaecology, University College of Medical Sciences, Delhi University, New Delhi, India

**Keywords:** Female factor, Hystero-laparoscopy, Hysterosalpingography, Infertility, Multidetector CT

## Abstract

**Background:**

Infertility is a common health problem requiring imaging to delineate the anatomical causes in women. Three dimensional multi-detector computed tomography hysterosalpingography (3D-MDCT-HSG) offers an easy workup for uterine, tubal and peritoneal factors.

**Methods:**

To present the spectrum of uterine, tubal and peritoneal factors on 3D-MDCT-HSG and determine its diagnostic accuracy for female factor infertility, a prospective study was conducted on 25 infertile women with non-diagnostic HSG from November 2012 to March 2014. Sixty four slice MDCT acquired the scan during pre-ovulatory phase by contrast instillation into uterine cavity. A blinded reviewer interpreted the 3D-MDCT-HSG and results were compared with final diagnosis made on hystero-laproscopy in 22 patients. Diagnostic accuracy of 3D-MDCT-HSG for various factors was expressed as sensitivity, specificity, positive and negative predictive value.

**Results:**

MDCT-HSG demonstrated definite findings in 96% of patients having non-diagnostic HSG. In this study, tubal, uterine and peritoneal abnormalities were present in 68.75%, 56% and 32% of cases, respectively. 48 tubes in 25 patients were evaluated of which 22 tubes were blocked constituting the commonest finding present in 15 (60%) patients. The sensitivity, specificity, positive predictive value and negative predictive value for uterine factors was 83.33%, 100%, 100% and 96.84%, respectively, for tubal factors 93.55%, 94.68%, 85.29% and 96.83%, respectively and for peritoneal factors 62.5%, 92%, 71.43% and 88.46%, respectively. Mean effective radiation dose was 1.76±0.18 *mSv* in MDCT-HSG.

**Conclusion:**

3D-MDCT-HSG can detect various factors responsible for female infertility especially tubal and uterine; in cases where HSG fails to clearly delineate the pathology.

## Introduction

Infertility is a great health and financial burden for the society. Its prevalence is estimated as 3.9 to 16.8 percent in different regions of India (1). World over, every one in six couple is infertile (2). Both functional and anatomical causes may be responsible and anatomical causes include uterine, fallopian tube and peritoneal factors each affecting one third of cases. Therefore, a comprehensive and accurate imaging of the patho-anatomy of female reproductive tract is required to detect the specific morphological factor responsible for female infertility (3).

The imaging armamentarium includes trans-abdominal sonography (TAS) and trans-vaginal sonography (TVS), hysterosalpingography (HSG), sonohysterosalpingography, saline/contrast infusion sonohysterosalpingography and Magnetic Resonance Imaging (MRI). At present, no modality is free from limitations and hence multiple techniques are utilized for evaluation of entire female reproductive tract (3–9). Selection of modality is often confusing, and use of multiple techniques increases cost and time of the diagnostic work-up and also results in duplicity of imaging information. Moreover, requirement of multiple hospital visits is discouraging for the poor patients earning on day to day basis and also for women in conservative societies.

Therefore, there is a need for an imaging modality which can evaluate all the three morphological factors of infertility in a single setting and with reasonable diagnostic accuracy.

Traditionally hysterosalpingography (HSG) is the basic radiological modality utilized for evaluation of the uterine (endometrial cavity), tubal and peritoneal factors. Hysterolaparoscopy though invasive is the gold standard investigation. Hysteroscopy provides direct visualization of the uterine cavity and is easily performed in an out-patient setting (14) while, laparoscopy directly visualizes fallopian tubes, assesses patency by chromotubation and the peritoneum by direct inspection (8).

Three dimensional multi-detector computed tomography hysterosalpingography (3D-MDCT-HSG) is a new hybrid modality involving imaging of contrast filled uterine and tubal lumen and the peritoneal spill using a MDCT. As CT has greater sensitivity for contrast it better visualizes the contrast filled tubes without the problems of overlapping or obscuration of tubes by peritoneal spill or inadequate opacification due to reflex cornual spasm as in HSG (10, 11).

Acquisition of thin slices allows post-processing to provide multiplanar reformatted (MPR) images to evaluate external contour of uterus as well. Assessment of the peri-tubal and peritoneal region non-invasively is an added advantage permitting detection of the peritoneal factor. Thus, 3D-MDCT-HSG provides a comprehensive evaluation of entire reproductive tract in a single setting (12, 13).

The purpose of this study was to evaluate the role of 3D-MDCT-HSG in infertile women with the objectives to present the spectrum of uterine, tubal and peritoneal findings and to assess the diagnostic accuracy (sensitivity, specificity, positive predictive value and negative predictive value) for detection of uterine, tubal and peritoneal factors of infertility.

## Methods

After obtaining due clearance from the institutional ethical committee and a written informed consent from the patients, a “prospective study” was conducted from November 2012 to March 2014, on infertile women referred to department of Radiology for an HSG examination.

### Cases:

25 consecutive patients showing inadequate, indeterminate or equivocal findings on HSG were included in the study and subjected to MDCT-HSG examination using a standard protocol. Patients refusing to give consent, having active pelvic infection or possibility of pregnancy were excluded.

### Imaging:

MDCT-HSG was acquired during the pre-ovulatory phase (seventh to tenth day of the menstrual cycle) using a 64 slice (Somatom Definition AS, Siemens AG, Germany) MDCT scanner by MS. Axial sections of 1 *mm* thickness and 64×0.6 *mm* collimation and reconstruction interval of 0.4 *mm* were obtained with 120 *kV* and reference milliampere of 200 *mAs*. 20 *mg* of hyoscine butylbromide was administered intramuscularly 20 *min* prior to CT examination. The patients lay in a supine position with their feet towards the gantry. The perineum was cleansed with 1% povidone iodine and foley’s bulb was inflated in the cervical canal. 15 *ml* of diluted non-ionic contrast was instilled through foley’s at 0.3 *ml/s* using a pressure injector and scanning done after 35 *s*. Patients were observed for 30 *min* post-CT for any complication. Analgesics were given to patients complaining of pain and prophylactic antibiotics were prescribed in all.

### Post processing and image analysis:

The acquired data was transferred to the work station and post processing done to obtain multi-planar reformatting (MPR), maximum intensity projection (MIP) and volume rendered (VR) images. 3D thick MIP images delineated the contrast filled endometrial cavity and fallopian tubes. VR provided a three dimensional view of endometrial cavity and tubal lumen. Images were reviewed by the blinded radiologist (SB), findings recorded and 3D-MDCT-HSG diagnosis made.

The imaging diagnosis was categorized as uterine, tubal and peritoneal and revealed to the clinician after the hystero-laparoscopy had been performed and findings were recorded. The hystero-laparoscopy was performed by the clinician (GR) within 1 to 2 months of MDCT-HSG, and tissue samples were taken wherever necessary and a final diagnosis was obtained in 22 patients.

Two patients had a unicornuate uterus, therefore MDCT-HSG evaluated a total of 48 tubes in 25 patients and evaluation for uterine and peritoneal factors was done in all.

Mean effective radiation dose in MDCT-HSG was calculated from the dose length product (DLP) using conversion factors from National Board of Health, National Institute of Radiation Hygiene, Denmark.

### Statistical analysis:

Data was analyzed by statistical software SPSS version 20. The various uterine and peritoneal factors (findings and diagnosis) were expressed as percentages per 25 cases. Fallopian tube findings were expressed as percentage of tubes (n=48 tubes).

Results obtained by MDCT-HSG were compared with hystero-laparoscopy and diagnostic accuracy calculated using contingency tables. Results for various factors and individual pathologies (wherever possible) were expressed as sensitivity, specificity, positive predictive value (PPV) and negative predictive value (NPV).

## Results

### Study population:

The study consisted of 25 female patients, age ranged from 20 to 37 years with mean age of 27.38 years. Thirteen (52%) had primary and twelve (48%) patients had secondary infertility.

3D-MDCT-HSG was successfully conducted in all except one patient. The uterine cavity was adequately distended and delineated in all patients. Fallopian tubes were evaluated in all except one patient because of non-opacification of tubes due to technical failure. Peritoneum was evaluated if contrast spill occurred from either or both tubes. MDCT-HSG demonstrated definite findings in 24 out of 25 (96%) patients while HSG was non-diagnostic in all these patients and thus selected for the study. The comparison of 3D-MDCT-HSG diagnosis with final diagnosis made on Hystero-laproscopy was done in 22 patients.

### MDCT-HSG findings and diagnosis:

Based on 3D-MDCT-HSG, 23 (92%) patients had at least one abnormal finding. In two patients, no factor was identified. A uterine factor was present in 14 (56%) patients, tubal in 19 (76%) and peritoneal in 10 (40%) patients (Table 1). The commonest uterine, tubal and peritoneal finding was abnormal shape of the endometrial cavity observed in seven (28%) patients, a blocked tube in 22 (48.83%) out of 48 tubes and loculated spill in 6 (24%) patients, respectively (Table 2).

**Table 1.** Spectrum of uterine (n=25), fallopian tube (n=48) and peritoneal (n=25) abnormalities on 3D-MDCT-HSG

**Uterine findings**	**Number of patients**	**Percentage**
**Endometrial cavity**		
**Abnormal shape**	7	28%
Small spindle shaped cavity	2	8%
Two separate uterine cavities	2	8%
Smooth indentation in the fundal region	3	12%
**Abnormal size**	5	20%
Large uterine cavity	2	8%
Small uterine cavity	3	12%
**Filling defect**	4	16%
**Irregular outline**	3	12%
**Abnormal External contour**	2	8%
**Enlarged uterus**	1	4%
**Extravasation of contrast into myometrium**	1	4%
**Tubal Findings**	No. Of tubes	Percentage
Visualized tube with free intra-peritoneal spill	22	45.83%
Tubal block	22	45.83%
Dilated fallopian tube	14	29.17%
Irregular outline^1^	10	20.83%
Extravasation of contrast^1^	2	4.17%
Beaded appearance^1^	4	8.33%
Thickened wall^1^	2	4.17%
Thickened tube^1^	1	2.08%
**Peritoneal findings**	Number of patients	Percentage
Loculated spill	6	24%
Nodules (as filling defect in spilled contrast)	1	4%
Calcification	0	0
Ovarian enlargement	4	16%
Prominent parametrial vessels	1	4%
Free fluid in pouch of duglas	1	4%

1: suggestive of salpingitis

**Table 2.** 3D-MDCT-HSG diagnosis in 25 patients

**Uterine factor**	**Number of patients**		**Percentage**	
**Congenital uterine malformation**	7		28%	
Unicornuate (Type II)	2		8%	
Septate (Type V)	2		8%	
Arcuate (type VI)	3		12%	
**Uterine fibroids**	4		16%	
**Endometritis**	3		12%	
**Intrauterine Adhesion**	2		8%	
Tubal factor (no of patients=25, tubes= 48)	Patients	Tubes	Patients	Tubes
Tubal Block (U/L^1^=8, B/L^2^=7)	15	22	60%	48.8%
Hydrosalpinx (U/L=4, B/L=5)	9	14	36%	29.17%
Salpingitis (U/L=1, B/L=6)	07	13	28%	27.08%
Peritoneal Factor	Number of patients		Percentage	
Peritubal adhesions	10		44%	
Enlarged ovaries	4		16%	
Endometriosis	1		4%	
PID^3^	1		4%	

1 - Unilateral,

2 - Bilateral &

3 - Pelvic inflammatory disease

Various MDCT-HSG diagnoses are depicted in table 2. The commonest 3D-MDCT-HSG diagnosis was tubal block seen in 22 tubes (48.8%) in 15 (60%) patients. Peri-tubal adhesions were present in 10 (40%) patients followed by hydrosalpinx in 13 (27.08%) tubes in 9 (36%) patients, salpingitis and uterine malformation in 7 (28%) patients each.

The diagnostic accuracy of MDCT-HSG for various factors detected in 22 patients is shown in table 3.

**Table 3.** Diagnostic accuracy parameters of 3D-MDCT-HSG for detecting various uterine, tubal and peritoneal abnormalities are also shown

**No of patients & Factors**	**TP**	**FP**	**FN**	**TN**	**Sensitivity**	**Specificity**	**NPV**	**PPV**
**Uterine (n=22)**								
Uterine malformation	7	0	0	15	100%	100%	100%	100%
Endometritis	3	0	1	18	75%	100%	94.7%	100%
Intrauterine adhesions	3	0	1	18	75%	100%	94.7%	100%
Fibroids	2	0	0	20	100%	100%	100%	100%
Polyps	0	0	1	21	0	100%	0	95.4%
**Fallopian tube (n=42 tubes)**								
Tubal block	13	4	0	25	100%	86.2%	100%	76.4%
Hydrosalpinx	11	1	1	29	91.6%	96.6%	96.6%	991.6%
Salpingitis	5	0	2	35	71.4%	100%	94.5%	100%
**Peritoneal (n=22)**								
Peritubal adhesion	5	4	1	12	83.3%	75%	92.3%	55.5%
Endometriosis	1	0	3	18	25%	100%	85.7%	100%
Enlarged ovaries	4	0	2	16	66.6%	100%	88.8%	100%

TP-true positive, FP- false positive, TN-true negative, FN-false negative, Sen-sensitivity, Sp-specificity, NPV-negative predictive value & PPV-positive predictive value

### Uterine factors:

**Figure 1.**
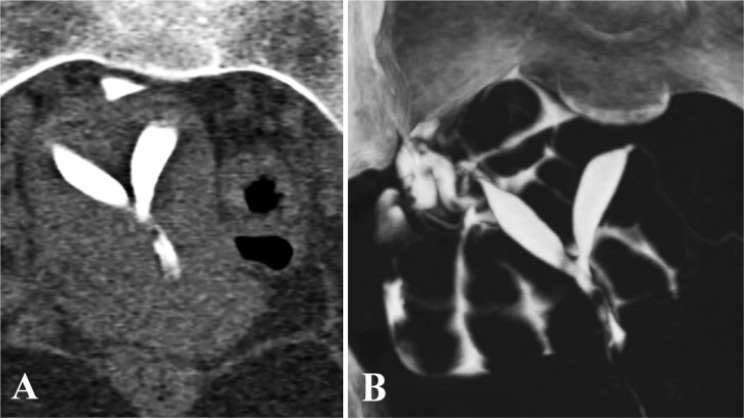
A: MPR coronal image shows incomplete uterine septum separating the two spindle shaped cavities, uterine fundal contour appears normal. B: MIP image showing septate uterus

All seven cases of congenital uterine malformation were correctly identified on 3D-MDCT-HSG. Most common uterine findings were arcuate uterus, found in 42.85% patients. Other malformations encountered were subseptate and bicornuate uteri, which were present in 28.57% patients (Figures 1A and 1B).

**Figure 2.**
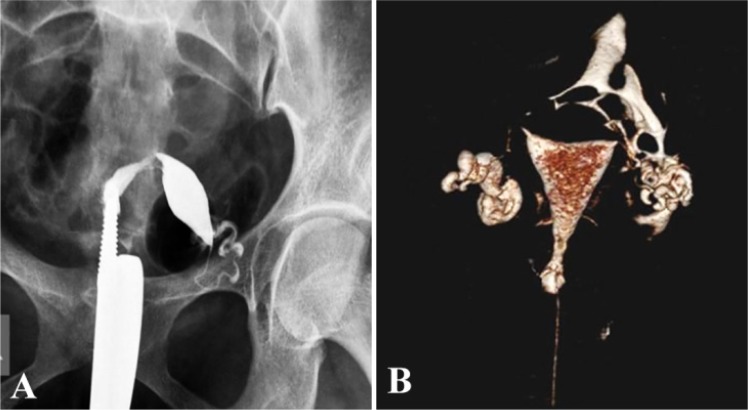
A: Abnormal uterine position on HSG shows a spindle shaped uterine cavity deviated towards left with patent left fallopian tube. Right fallopian tube is not seen. Findings are suggestive of a unicornuate uterus on HSG. B:VRT image from MDCT-HSG shows a normal uterine cavity, right terminal hydrosalpinx with fimbrial block, left fallopian tube is patent. However, non-filling of right tube led to the misinterpretation of a unicornuate uterus on HSG

A retroverted uterus was identified in five patients of which one patient in addition demonstrated rotation of the retroverted uterus with bilateral patent tubes (Figures 2A and 2B) while on HSG it appeared as a unicornuate uterus.

**Figure 3.**
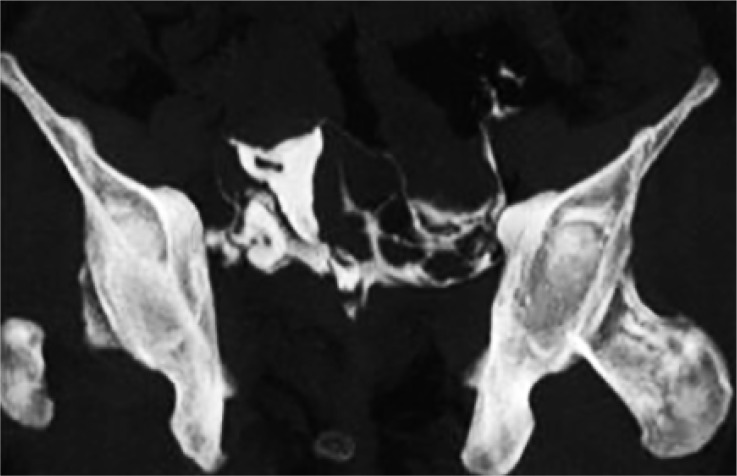
MIP image from a patient shows evidence of linear filling defect within the contrast filled uterine cavity suggestive of intrauterine adhesion

**Figure 4.**
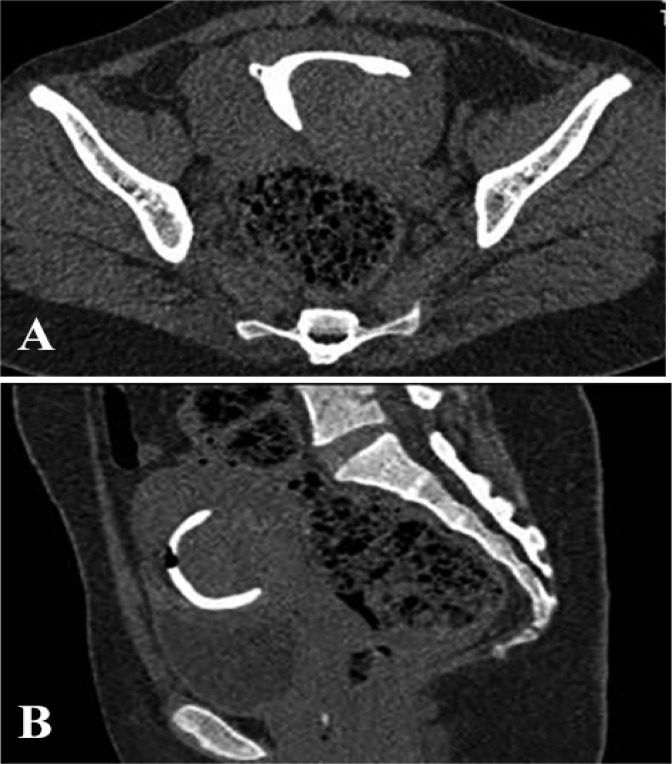
A: Axial and figure B: Sagittal MPR image show a large broad based postero-lateral filling defect within the contrast filled uterine cavity suggestive of a submucosal leiomyoma

Endometritis appeared as an irregular endometrial outline, extravasation of contrast, irregular filling defect or abnormally small cavity on MDCT-HSG. 75% patients of endometritis were detected on 3D-MDCT-HSG. Similarly, 75% patients of intrauterine adhesions were correctly identified (Figure 3) and only one (25%) was missed. A single case of a submucosal polyp was also missed on 3D-MDCT-HSG. MDCT-HSG correctly picked-up all the fibroids in the study. It detected the submucosal fibroid (Figure 4A and 4B) as well as subserosal, intramural fibroids, which were missed on HSG examination.

### Fallopian tube factors:

**Figure 5.**
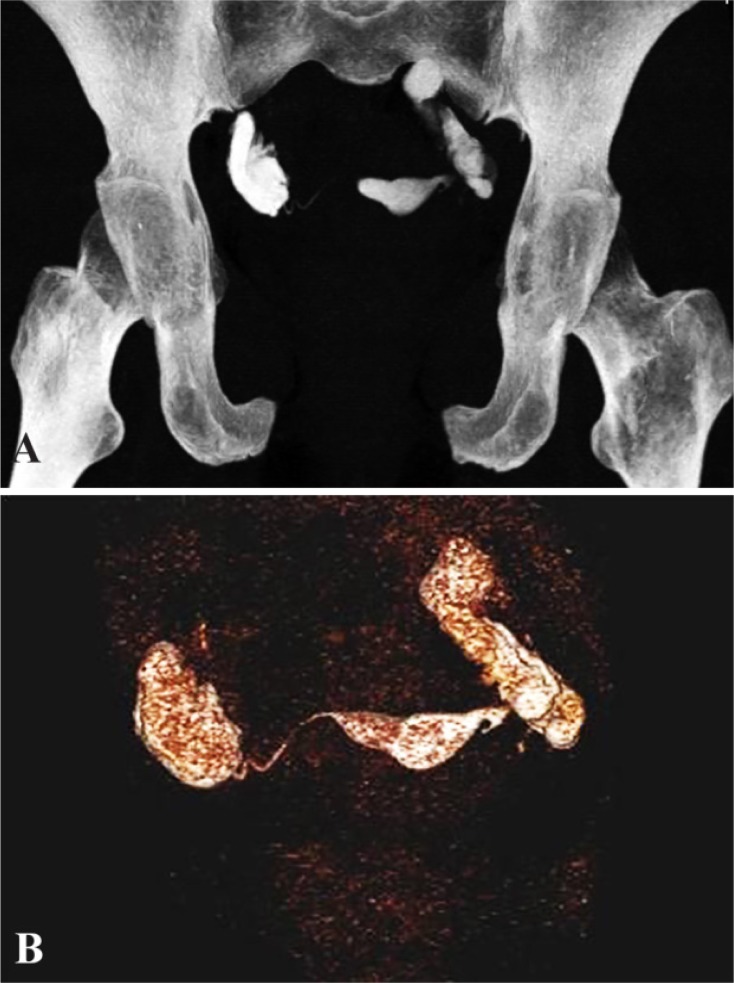
A: MIP and figure B: VRT image showing bilateral hydrosalpinx with minimal spill on right side

**Figure 6.**
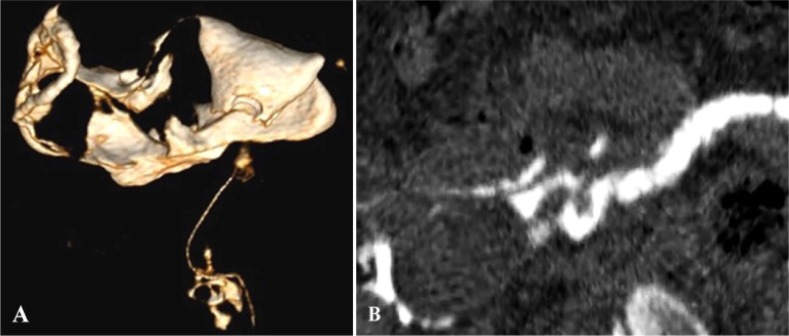
A: VRT shows normal uterus with patent right fallopian tube with peritubal spill and left tubal block; B: Curved MPR excellently unfolds the convoluted right fallopian tube which shows evidence of beading and irregular outline suggestive of salpingitis. Right ovary is seen as an ovoid filling defect within the spilt contrast.

On laparoscopy, 30.95% of tubes were found to be blocked and MDCT-HSG correctly identified all blocked tubes. Tubal occlusion appears as non opacification or abrupt cut off of the contrast column in the tube with absence of contrast spillage (Figure 6A). In addition, four tubes were falsely positive. Hydrosalpinx was detected in 12 out of 42 (28.57%) fallopian tubes on laparoscopic examination, of which 11 tubes were correctly identified on 3D-MDCT-HSG as a dilated tube (Figures 5A and 5B). Salpingitis appeared as irregular outline of tubes, beaded appearance, thickened walls and thickened fallopian tube (15, 16) (Figure 6B). Extravasation of contrast was a new finding recognized on MDCT-HSG. Five out of seven tubes were correctly diagnosed on MDCT-HSG while two patients were missed. MDCT-HSG detected salpingitis in 31.8% as opposed to 18.2% on HSG.

### Peritoneal factors:

Six patients had peritubal adhesions on laparoscopy while MDCT-HSG made this diagnosis in 9 patients (36.36%). Diagnostic criteria used for peritubal adhesion on MDCT-HSG was based on presence of one or more findings of convoluted fallopian tube, loculation of contrast, ampullary dilatation, peri-tubal halo effect and vertical fallopian tube. Four patients were found to be falsely positive and one patient was missed on MDCT-HSG.

**Figure 7.**
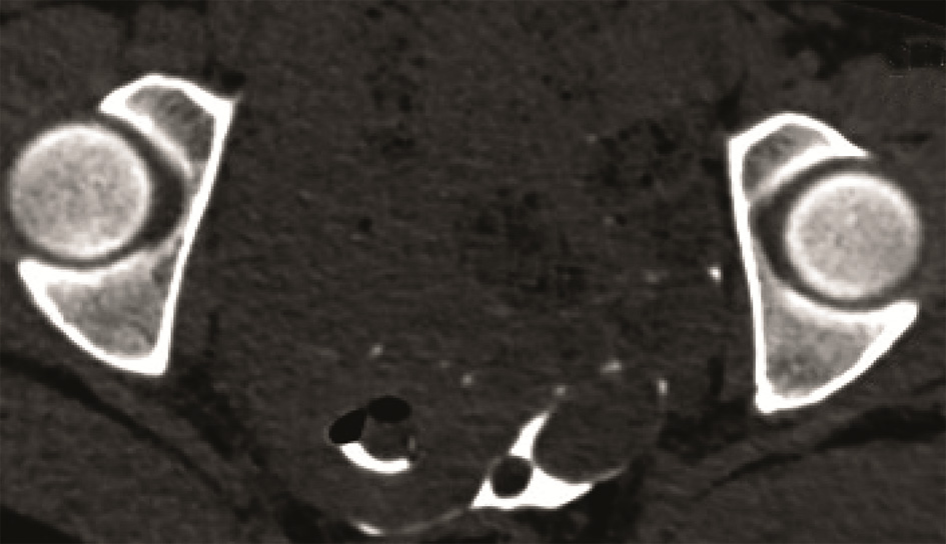
MIP image shows the left ovary as an oval structure delineated by the contrast spill. A round soft tissue density lesion noted postero-medial to the ovary was confirmed as an endometriotic deposit on laparoscopy

Enlarged ovaries were detected in six patients on laparoscopy of which one had a tubo-ovarian mass. MDCT-HSG detected four (66.7%) cases out of six positive cases. Three cases of endometriosis and one of peritoneal tubercles were identified on laparoscopic examination, out of which only one case of endometriosis was detected on 3D-MDCT-HSG. This was noted as a nodular filling defect on the surface of the ovary delineated by the intra-peritoneal spill (Figure 7). Comparison of 3D-MDCT-HSG diagnosis with final diagnosis made on Hystero-laproscopy in 22 patients.

One patient was normal and 21 had one or more positive findings. Seven patients had presence of only one factor of infertility, 9 had presence of 2 factors while 5 women had presence of all the 3 uterine, tubal and peritoneal factors of infertility. Identification of all factors on MDCT-HSG was considered complete diagnosis and if any factor was missed, the diagnosis was considered incomplete. A complete diagnosis was made in 14 out of 22 (63.63%) patients of which one was normal. An incomplete diagnosis was made in seven (31.81%) patients and in one (4.54%) patient the diagnosis was missed. Additional findings were noted in eight patients on 3D-MDCT-HSG. The factors missed on MDCT were peritoneal (4 patients- 3 peritoneal nodules and 1 peri-tubal adhesion), intrauterine (3 patients-1 mild endometritis, 1 small cervical submucosal polyp, 1 intrauterine adhesion) and tubal (1 patient of hydrosalpinx). In four patients, additional findings were picked up on MDCT-HSG but were clinically significant in only one patient showing hemorrhagic cyst in the ovary.

Mean effective radiation dose in MDCT-HSG was 1.76±0.18 *mSv*.

## Discussion

25 patients selected on the basis of non-contributory HSG underwent MDCT-HSG which demonstrated definite imaging findings and provided an MDCT-HSG diagnosis in 24 patients (Table 2). Therefore, MDCT proved superior to conventional HSG in 24 out of 25 (96%) patients where the anatomical cause for female infertility could be demonstrated.

On comparison with gold standard hystero-laproscopy in 22 patients, a complete diagnosis (detection of all the factors) was made in 14 out of 22 (63.63%) patients of which one was normal. An incomplete diagnosis (not all the factors detected) was made in seven (31.81%) patients and in one (4.54%) patient, the diagnosis was missed.

Female factors responsible for infertility can occur in isolation or in various combinations. Seven patients had presence of only one factor of infertility, 9 had presence of 2 factors while 5 women had presence of all the 3 uterine, tubal and peritoneal factors of infertility. Therefore, MDCT–HSG is able to evaluate all the three uterine, tubal and peritoneal factors in a single setting. There was a high incidence of tubal block (60%) in our study, and this may be due to pelvic inflammatory disease (PID) being the most common gynaecological disease (17).

The sensitivity, specificity, positive predictive value and negative predictive value for uterine factors was 83.33%, 100%, 100% and 96.84%, respectively, for tubal factors 93.55%, 94.68%, 85.29% and 96.83%, respectively and for peritoneal factors 62.5%, 92%, 71.43% and 88.46%, respectively, giving a reasonably good diagnostic performance for the uterine and the tubal factors. However, for the peritoneal factors, the diagnostic performance was low. Each factor is discussed in detail.

### Uterine factors:

The sensitivity and negative predictive value was 83.33% and 96.84%, respectively for uterine pathologies collectively as opposed to 100% in previous study (18).

This can be attributed to the use of cervical cannula in the other study instead of a balloon catheter as in ours, posing no hindrance to visualisation of the intra-uterine abnormalities. Virtual endoluminal evaluation was also performed in their study, which clearly facilitates the identification of intra-uterine lesions such as polyps or submucous myomas as well as intrauterine adhesions. However, the specificity and PPV were 85.71% and 84.61%, respectively in their study as opposed to 100% in the present study. This could be because of initial use of MDCT (16 slice) in evaluation of the female reproductive tract versus 64 slice used after approximately seven years in our study resulting in better delineation of abnormalities due to better image resolution and experience of the technique.

MDCT-HSG had a diagnostic accuracy of 100% for detecting fibroids and congenital uterine malformations. Sensitivity, specificity, NPV and PPV were all 100%. Arcuate uterus was found in 42.85% of patients and was also the most common malformation noted by other authors (19, 20). The reformatted images depicted the uterus were convincing thus they prevented misinterpretations which may occur in HSG unless additional views are taken. Demonstration of a fundal cleft on the MPR images helps to distinguish a bicornuate from a septate uterus. Capability of assessing external contour along with the uterine cavity on MDCT-HSG is an added advantage over other imaging techniques for achieving a precise diagnosis. 3D-MDCT-HSG provides an enhanced appreciation of the spatial relationships of uterus with other pelvic structures. The results of our study were congruent with literature in this respect (18).

MDCT-HSG had a high specificity (100%) but moderate sensitivity (75%) in identifying cases of endometritis and intrauterine adhesions (Figure 3) with positive predictive value being 100% and negative predictive value being 94.75%.

Subtle uterine cavity findings like endometritis may be missed on imaging studies even MDCT-HSG as happened in our study. However, on careful retrospective review a small linear irregular filling defect was identified on the MIP images, which suggested the presence of an intrauterine adhesion. To the best of our knowledge, no literature is available on MDCT-HSG with regard to endometritis. Limitation in detecting intra-uterine pathology such as intrauterine adhesion was also mentioned by previous authors with 64-row multi-detector CT virtual hysterosalpingography (12). Intrauterine pathology may be missed when subtle as in our study or become obscured if it is present adjacent to the distended foley’s bulb. A small submucosal polyp missed in our study on 3D-MDCT- HSG was due to the presence of the foley’s balloon high within the uterine cavity. However, virtual MDCT-HSG has a better diagnostic evaluation for intrauterine filling defect and can confidently differentiate a polyp from a submucous myoma (18). Only submucosal fibroids which project into the uterine cavity and are seen as filling defect can be detected on HSG. Subserosal or intramural fibroids until calcified or large enough to enlarge or distort the uterine cavity are not apparent on HSG examination. However, all were identified on MDCT-HSG in our study as it allowed evaluation of both the uterine contour as well as the contrast filled uterine cavity as also observed by Carrascosa et al. (12, 13). Though evaluation of the size, location and depth of submucous myomas presenting as intrauterine filling defect can be achieved on MDCT virtual HSG but it is better to be delineated for giving intravenous contrast (18).

### Fallopian tube factors:

The sensitivity, specificity, positive predictive value and the negative predictive value for all the tubal factors collectively was 93.55%, 94.68%, 85.29% and 96.83%, respectively, which was less than 100% in the study conducted by Carrascosa et al. (18).

The present study showed a perfect sensitivity and NPV of 100% in detecting tubal block by MDCT-HSG, which was similar to other researchers (21). However, their study showed better specificity and PPV of 96.70% and 83.30% as against 86.20% and 76.40% in our study, respectively. The experience of the radiologist interpreting the MDCT-HSG scan may have accounted for this difference. MDCT-HSG detected hydrosolpinx with a better sensitivity than salpingitis (91.6% *vs*. 71.4%), while the specificity was more for detection of salpingitis (100% *vs*. 96.6%).

CT is more sensitive to detect contrast and therefore has a better performance in delineating the fallopian tubes. Better appreciation of the contrast filled tubes accounted for better results of 31.8% on MDCT-HSG as opposed to 18.2% on conventional HSG. In addition, axial CT images help in differentiating tubes from the spilled contrast at times of confusion (13). Associated extravasation of contrast from the tubes was an important finding noticed in our study in two patients suggesting inflammation of the tubes. Failure of opacification of the tube may occur if insufficient relative pressure is generated due to use of pressure injector rather than hand injection.

### Peritoneal factors:

Peritubal adhesions occur secondary to previous surgery, infection or inflammation similar to the cause of tubal occlusion. The sensitivity and negative predictive values (83.3% and 92.3% respectively) were more than the specificity and positive predictive value (75% and 55.5% respectively) of MDCT-HSG in diagnosing peritubal adhesions. All patients showing false positive results demonstrated ampullary dilatation with contrast spill as also concluded by Karasick and others that ampullary dilatation in non-occluded tubes cannot be reliably used to make diagnosis of peritubal adhesions (22). Tubal blockage not allowing contrast to reach the peritoneum limits the usefulness of MDCT in diagnosing peritubal adhesions as in our study.

For detecting peritoneal endometriosis, 3D-MDCT HSG has a high specificity and positive predictive value of 100% but a low sensitivity (25%) and negative predictive value (85.7%), respectively. This is because peritoneal lesions are better appreciated on laproscopy by direct inspection than being assessed indirectly against spilt peritoneal contrast on MDCT-HSG. Lack of intra-peritoneal spillage of contrast with tubal blockage/peritubal adhesions did not allow detection of peritoneal lesions giving high false negative rate. MDCT-HSG does not seem to be an attractive modality for peritoneal abnormalities causing infertility in females. These patients require diagnostic laparoscopy for effective management.

Ovarian enlargement was detected with a sensitivity of 66.7% and specificity of 100%. The PPV was 100% while the NPV was 88.8%. For ovarian pathology, a significant time gap of 1 to 2 months between CT and hystero-salpingography may account for the low sensitivity on MDCT-HSG in this study. Flaring up of the pathology in the intervening period resulted in a larger number of patients showing ovarian pathology on laparoscopic examination.

Majority of the factors missed on MDCT were peritoneal and occurred due to lack of peritoneal spill. The overall sensitivity, specificity, positive predictive value and the negative predictive value for the peritoneal factors was low and was 62.5%, 92.0%, 71.43% and 88.46%, respectively.

Mean effective radiation dose of 1.76±0.18 *mSv* in MDCT-HSG in this study was lower than 2.58± 0.75 *mSv* (12) and 3.02±0.15 *mSv* (21) found in two previous studies. A slightly less effective radiation dose of 1 *mSv* has been reported for conventional HSG, however, it is extremely difficult to achieve this low radiation dose in daily practice (23). On the other hand, there is negligible risk of radiation exposure to the radiology staff at MDCT-HSG in comparison to HSG.

The limitations of the study were a relatively small number of patients, non-administration of intravenous contrast and use of foley’s catheter with its bulb inflated in the cervix limiting evaluation of cervical canal.

## Conclusion

Overall, 95.45% patients were diagnosed (either complete 63.63% or incomplete/partial 31.81% patients) on MDCT-HSG even though the diagnosis was inconclusive on HSG in all these patients. 3D-MDCT-HSG allows complete morphological assessment of the anatomy of female reproductive tract and assessment of all the three factors of infertility due to an anatomic cause. It has a good performance in detecting uterine and tubal factors of infertility but its use in peritoneal factors is limited. This is an extremely useful modality rendering a low radiation dose and showing promising initial results, also in patients with non-diagnostic HSG.

### Clinical relevance:

Due to its reasonable accuracy, non-invasive nature unlike hystero-laproscopy and capability to provide complete assessment in a single setting, even in situations where HSG is non-contributory it has the potential of becoming an initial screening modality for evaluation of the morphological factors of female infertility or it can be used as a diagnostic modality where HSG fails or when hystero-laproscopy is not preferred by the patients.
